# Before the Lords' Committee

**Published:** 1890-08-09

**Authors:** 


### Before the Lords' Committee.

(By Our Special Commissioner.)

### X.—THE LAST OF THIS SESSION.

The last meeting for this Session of the Select Committee on Metropolitan Charities was held on July 31st. Only five of the noble lords were present, and the audience was small; evidently the interest in the subject was decreasing. The first witness called was Mrs. B. Fenwick, late matron of St. Bartholomew's. She gave it as her opinion that there should be a sub-committee on nursing in every large hospital, to consist of medical and lay men, but professed she would be very sorry to be in any way connected with a committee of women. In a model ward of thirty beds Mrs. B. Fenwick would have, as nursing staff for the day, one sister, one staff nurse, one senior probationer, three junior probationers, and one ward maid; for night duty, one staff nurse, one senior probationer, and one junior probationer. There used to be a great deal of sickness amongst the nurses of St. Bartholomew's, but during the last ten years the staff has been doubled, and the death-rate has decreased. Considering the education they receive, probationers should pay both in time and money for their training as nurses. Asked about registration, Mrs. B. Fenwick said six of the large London hospitals were in favour of it; asked the names of the six, she replied, after some hesitation, St. Bartholomew's, Guy's, Middlesex, University, and formerly the Royal Free. At the best, this only makes four, and our readers must be perfectly aware that Dr. Norman Moore and Mr. Harrison Cripps, instructors of nurses at St. Bartholomew's, and Dr. Perry, Dr. Newton Pitt, and Dr. Steele, instructors of nurses at Guy's, have signed the great protest against registration. Lord Cathcart called Mrs. B. Fenwick's attention to this, and read the protest and the long list of schools that have signed it, including St. Thomas's and the Nightingale Fund, Charing Cross, Westminster, The London, King's College, St. Mary's, St. George's, St. Marylebone, The Royal Free, St. Pancras, and about 65 metropolitan and provincial nursing schools. These sounded rather convincing against Mrs. B. Fenwick's doubtful four.

Lord Cathcart rather raised a laugh then by stating that he understood a nurse was composed of two parts: one part female medical student, and one part ministering angel; and that no register could record the qualities of the ministering angel, which can only be understood by those who oversee a nurse's daily work.

Lord Kimberley asked if a central register would not be likely to produce extraordinary embarrassment and difficulty amongst the vast number of people who nurse amongst the poor; for a nurse who trained three years could not afford to take the very small sum the poor could offer her. Mrs. B. Fenwick replied: "There is no reason why a nurse engaged in that portion of the work should be registered." Asked what penal powers the board of registration would have, Mrs. Fenwick said: "They would have no powers of legal prosecution if a nurse contravened any of the laws laid down." Your mystified Special Commissioner wondered what on earth was the use of a register which did not include all nurses, and had no powers to enforce its laws. Would it alter in the least the present state of things? And if so, how?

Sir Andrew Clark, president of the Royal College of Physicians, was next called, and enlivened the proceedings by his interesting and anecdotal style of giving evidence. Dr. Gilbart Smith and other members of the medical profession came into the room for this part of the proceedings. Sir Andrew stated he was first connected with the London Hospital in 1873, and retired about eighteen months ago; he had also had experience at Haslar and the Victoria Park Hospital. He considers that the shutting up of the out-patient department of the hospitals would be the greatest calamity. Medicine is an art and must be learnt as an art. It cannot be learnt from the reading of books. It is necessary that disease should be discovered in its early stages, and this can only be done in the out-patient department. The prognosis of a physician who has had no out-patient experience generally is bad; for instance, a celebrated physician became notorious for telling every patient who came to him with a little damage of the lungs or kidneys that he could not recover. We have also to bring up others to carry on our work when we are gone, and this cannot be satisfactorily done in the wards only, where disease is seen at its climax, not at its commencement. Sir Andrew said he never found it exceed his capacities to deal with the cases of the out-patient department, though he had no assistants; but the capacity for work seems to be diminishing in the men of to day. There is no serious abuse of the out-patient department, in illustration of which theory Sir Andrew told the story of a clergyman who once presented himself, and who had a wife and six children, and an income of £150 a-year. "The artizan earning 7s. 6d. a-day was rich compared to this clergyman," concluded Sir Andrew. With regard to the alleged overcrowding, Sir Andrew had complained occasionally when he found an extra bed in his ward, but he had nothing adverse to say. The nursing was excellent at the London; he often asked the patients if they were comfortable, and only once received a complaint, which was on examination proved to be unfounded. With regard to medical education, Sir Andrew had very strong views; he was in favour of general hospitals which attached medical schools. Special hospitals magnify complaints; a hospital is built for maladies of the big toe, they build up a history of the big toe, and a literature of the big toe, and the subject becomes a serious one. There is more publicity about a general hospital which is security for its trueness and thoroughness. The special departments of general hospitals can do all that is needed, save perhaps in the case of lying-in hospitals, children's, and eye hospitals. Cancer, consumption, and lock cases do not need special hospitals. Some amusement was caused by Lord Cathcart, who informed Sir Andrew he was a patron of the Hamilton Association of Male Nurses. Sir Andrew was evidently not aware of the fact, but accepted the statement, and said that though, speaking generally, he was in favour of female nurses, he had seen good results at Haslar, where there were male nurses. Attention was called to the fact that a patient in a lunatic asylum might have bronchitis, and be attended by an utterly untrained nurse. On the question of the relative value of medical degrees, Sir Andrew held that a man who aspired to be a hospital physician, and a teacher of students, should be bound to become a member of the Royal College of Physicians. The Edinburgh M.D. was a stiff examination; the examination questions of the London University were such that the lecturers themselves could not answer them; but the membership of the Royal College of Physicians proved that there was nothing against a man's moral character, and that he had the intellectual and scientific acquirements for teaching purposes. The infirmaries should be thrown open to students, both for the sake of the patients there, and of the teaching material. There should be a great central board to control matters common to all hospitals, but they should not be put under the State and made mere machines. A resident medical officer should be changed every five years; if he is a permanent official he becomes a nuisance, save at Guy's, where they have an exceptionally good man. With regard to teaching, the smaller medical schools, such as Charing Cross and Middlesex, might combine and get better lectures for such subjects as natural history, chemistry, &c.; but in practical subjects each student should be taught at the hospital to which he is attached.

